# Pandemic … or *syndemic*? Re‐framing COVID‐19 disease burden and ‘underlying health conditions’

**DOI:** 10.1111/1469-8676.12886

**Published:** 2020-05-19

**Authors:** Rebecca Irons

**Affiliations:** ^1^ Department of Anthropology University College London London WC1H 0BW UK

*What is in a name?* When the WHO declared coronavirus a global ‘pandemic’, it seems that this is when the world finally sat up to take serious notice of the impending threat as it gained momentum throughout the continents. Indeed, this change of term upgraded COVID‐19’s status from a faraway‐disease to something all‐encompassing and pan‐global. But importantly, it positioned the virus as something that should now concern ‘us’.

Infectious disease epidemics are not your everyday occurrence or concern if you live in a certain part of the world; they are arguably portrayed as a scourge of post‐colonial and so‐called ‘developing’ contexts, where a European and North American audience might suspect death and disease to be a regretful, if inevitable, part of historical and contemporary existence. ‘Epidemics’ and ‘disease’ happen ‘out there’. Zika took hold in the jungles of South America; Ebola ravages the civil‐war‐torn Congo; MERS infiltrated the insular Saudi peninsula; SARS erupted in Southern China. When cases arrived in Europe and North America, they were quickly contained, and the lay public had little cause for extended interaction with the news media about these epidemics.

However, COVID‐19 is different in an important sense: Europe, and now the USA, have become the epicentres for the virus, challenging post‐colonial perceptions that infectious‐disease epidemics are fundamentally a non‐‘Western’ concern. Alas, it would seem that for a disease to globally register as a ‘pandemic’, it does not necessarily need to include the whole world, but the part of the world that considers itself the most important. Certainly, reporters and observers have delighted in highlighting how COVID‐19 ‘does not discriminate’, and can *kill* individuals from across social strata and geographical locations. Yet, is this really true? Despite the sensationalist tabloids breathlessly reporting on the still‐rare deaths of younger people and those with no underlying health conditions, it remains that the vast mortality majority comes from the elderly and those who have pre‐existing health conditions. Coronavirus is no 1918 ‘Spanish’ Influenza that struck down a global population in its prime: this virus is overwhelmingly targeting people who would already be vulnerable to disease. From the available data, then, there is cause to suggest that this is not a *pandemic*, but a *syndemic*. A syndemic refers to the interaction of multiple epidemics that ultimately exacerbate the disease burden in certain populations and increase health vulnerability. Now, people with underlying health conditions and the elderly, whose immune systems may be weaker, are substantially more at risk from coronavirus. Significantly, multiple epidemic disease burdens are *not* of great threat to Europe and North America.

Here then, the Greek prefix *pan*‐, ‘all’ [of us], arguably does not work conceptually as a name for COVID‐19. This virus will not *corporeally* affect us ‘all’, but will disproportionately encumber those already burdened. Going forward, understanding this virus as a syndemic could contribute to more appropriate responses in our own communities, and globally. Importantly, it could potentially influence ‘selfish’ hoarding and quarantine‐flouting behaviours. From the name ‘pan*demic*’ leaps ‘pan*ic*’; re‐addressing the term may influence behaviours that save lives in the long run.

